# Gold Nanoparticle Self-Aggregation on Surface with 1,6-Hexanedithiol Functionalization

**DOI:** 10.3390/nano10030512

**Published:** 2020-03-11

**Authors:** Maksym Stetsenko, Tetiana Margitych, Serhii Kryvyi, Lidia Maksimenko, Ali Hassan, Svitlana Filonenko, Βaikui Li, Junle Qu, Elke Scheer, Sergii Snegir

**Affiliations:** 1 Key Laboratory of Optoelectronic Devices and Systems of Ministry of Education and Guangdong Province, College of Physics and Optoelectronic Engineering, Shenzhen University, Shenzhen 518060, China; stetsenkomax@gmail.com (M.S.); 15alirao@gmail.com (A.H.); 2 V. Lashkaryov Institute of Semiconductor Physics, National Academy of Sciences of Ukraine, 03680 Kyiv, Ukraine; serkriviy@gmail.com (S.K.); maximenko_lida@ukr.net (L.M.); 3 Kyiv Institute for Nuclear Research, National Academy of Sciences of Ukraine, 03680 Kyiv, Ukraine; margtanya@gmail.com; 4 Institute of Physics, Polish Academy of Sciences, 02-668 Warsaw, Poland; 5 Pisarzhevski Institute of Physical Chemistry, National Academy of Sciences of Ukraine, 31 Prospect Nauky, 03028 Kiev, Ukraine; svitmail@ukr.net; 6 University of Konstanz, Department of Physics, Universitätsstraße 10, 78464 Konstanz, Germany; Elke.Scheer@uni-konstanz.de

**Keywords:** aggregation, dimers, AuNPs, self-assembly, surface, 1,6-hexanedithiol, APTES, optics, morphology

## Abstract

Here we study the morphology and the optical properties of assemblies made of small (17 nm) gold nanoparticles (AuNPs) directly on silicon wafers coated with (3-aminopropyl)trimethoxysilane (APTES). We employed aliphatic 1,6-hexanedithiol (HDT) molecules to cross-link AuNPs during a two-stage precipitation procedure. The first immersion of the wafer in AuNP colloidal solution led mainly to the attachment of single particles with few inclusions of dimers and small aggregates. After the functionalization of precipitated NPs with HDT and after the second immersion in the colloidal solution of AuNP, we detected a sharp rise in the number of aggregates compared to single AuNPs and their dimers. The lateral size of the aggregates was about 100 nm, while some of them were larger than 1μm. We propose that the uncompensated dipole moment of the small aggregates appeared after the first precipitation and acts further as the driving force accelerating their further growth on the surface during the second precipitation. By having such inhomogeneous surface coating, the X-ray reciprocal space maps and modulation polarimetry showed well-distinguished signals from the single AuNPs and their dimers. From these observations, we concluded that the contribution from aggregated AuNPs does not hamper the detection and investigation of plasmonic effects for AuNP dimers. Meantime, using unpolarized and polarized light spectroscopy, the difference in the optical signals between the dimers, being formed because of self-aggregation and the one being cross-linked by means of HDT, was not detected.

## 1. Introduction

Gold nanoparticles (AuNPs) attract much attention owing to their unique optical, electronic, and catalytic properties. They have allowed development of different prototypes of devices for sensing [1,2], detecting [3], and imaging [4,5]. Further progress in designing AuNP-based devices depends on the ability to fabricate complex assemblies with tailored optoelectronic properties. Different approaches have already been proposed to follow this idea.

A multi-stage ligand exchange procedure [6,7,8] in a solution allows for the formation of monometallic dimers [9,10], trimers [11], long AuNP chains [12,13], or hybrid systems made of polystyrene-coated gold [14] or silver–gold [15] nanoparticles. However, during the synthesis, in addition to the dimers, larger structures with a different size and shape appear. Therefore, the process of the dimer separation becomes a complicated task. A DNA origami was used recently [8,16] to design more controlled self-assembled nanostructures with high yield. This approach allows for programmable spatial positioning of functionalized nanoparticles in a confined volume. As a result, 2D and 3D periodic assemblies of isotropic/anisotropic AuNPs with different spatial separation can be created. Self-assembly of AuNPs on a micro-scale requires exploiting their high affinity toward specific surface functional groups. Gold substrates coated with dithiols, for instance, can efficiently anchor AuNPs [17,18]. The surface of glass, silicon, and indium tin oxide (ITO) pre-coated with (3-aminopropyl)trimethoxysilane (APTES) can efficiently attach AuNPs [19,20,21] as well as NPs made of Ag [22,23], SiO_2_ [24], TiO_2_ [25,26], Fe_2_O_3_ [27,28], or Y-zeolites [29].

When an APTES-coated surface is used, a static interaction of the positively charged aminopropyl terminal groups and negatively charged cores of AuNPs, being stabilized by means of trisodium citrate in water, is employed. By changing the time of the substrate immersion in the colloid, the resulting surface density of single AuNPs can be adjusted. The contact point of the AuNPs with the substrate is quite small. Therefore, the remaining free AuNP surface can be further functionalized with different molecules either for studying the interaction of the localized surface plasmon resonance (LSPR) with their surrounding media [30] or for the synthesis of mono- and bi-metallic [31,32] NP dimers.

Thus, using APTES is the most straightforward approach for the immobilization of AuNPs on a large area. However, obtaining a coating with only single AuNPs well separated from each other is rather difficult to achieve because of the intrinsic presence of self-aggregated AuNPs. Their appearance is associated with the quality of the APTES coating [19]. The smoother the aminosilane monolayer is at the molecular scale, the lower will be the amount of aggregated AuNPs. Several approaches have been proposed to improve the quality of the APTES coating. At first, the substrate roughness has to be minimized [33,34]. Then, the surface density of the OH– terminal groups has to be sufficiently enhanced to be able to bind all three ethoxy groups of the APTES molecules. Then the optimal concentration of the APTES has to be used, and a suitable time of a substrate immersion has to be chosen [33]. Even with all of these steps, some ethoxy groups of the APTES can remain not completely linked to the surface. This can be improved partially by elevating the APTES solution temperature to ~70 °C during substrate silanization [35] or by post-annealing of the APTES-coated substrate at *T* = 600 °C in vacuum [19]. However, even with these treatments, finding the majority of the –NH_2_ groups of the APTES monolayer pointing away from the surface is difficult to achieve [36] and, thus, completely suppressing self-aggregation of AuNPs is impossible.

In our previous article [20], we developed and tested the method of solid-state formation of dimers created from small AuNPs (~18 nm) cross-linked with 1,9-nonadithiol (NDT) molecules on glass surfaces. In addition to the dimers, we detected the formation of the aggregates that led to the appearance of collective oscillation modes, which were self-similar to AuNP dimers. We concluded that the study of the optical properties of AuNP dimers on the surface requires further development.

Therefore, this work aims at creating cross-linked self-assemblies of AuNPs with a naturally formed amount of aggregates on silicon (Si) wafers to understand the possibility of detecting the optical signal from single AuNPs and their dimers. We used smooth silicon (Si) wafers instead of the glass [20] to minimize the number of defects in the APTES monolayer that might appear due to intrinsic wafer defects [33,34]. With atomic force microscopy (AFM), scanning electron microscopy (SEM), X-ray diffraction (XRD), and X-ray reflectivity (XRR), we studied at first the morphology of the cross-linked AuNP assembly, which was generated directly on the APTES-coated Si wafer. We used the conventional method of APTES deposition i.e., the immersion of an Si wafer in the APTES/methanol solution with subsequent drying. After the first immersion of this substrate in AuNPs colloidal solution, we achieved a certain amount of self-aggregated nanoparticles. Before the second immersion, we modified the AuNPs with 1,6-hexanedithiol (HDT), which by one –SH group attached to the first AuNP and by the opposite –SH to the second AuNP, forming the dimer. HDT represents a class of molecules that is commonly used as a crosslinking agent [6,7,37,38]. However, so far, it has not been sufficiently studied how well dithiols cover the curved surface of AuNPs, in particular their facets. On flat Au(111) surfaces, the length of the molecules can determine the structure of the formed self-assembled monolayer [39]. Therefore, we chose shorter HDT molecules, instead of longer NDT being used in earlier studies [20], to minimize the possibility of molecules lying flat on the surface with both –SH terminal groups on the same AuNP.

We studied the influence of the morphology of the molecular layer on the plasmon resonance excitation mechanisms and their polarization properties in assemblies using Fourier-Transform Infrared Spectroscopy (FTIR), UV–vis near-infrared spectroscopy, and modulation–polarization spectroscopy (modulation polarimetry) [40,41].

## 2. Materials and Methods

Formation of AuNPs dimers was performed following the procedure described in detail in [20]. In brief, Si wafers with native oxide and pre-coated with APTES (Merck, USA) were immersed into a water colloidal suspension of AuNPs for 30 min (in the following labeled as **S30**). Next, the wafer was first immersed in a solution of HDT (Merck, USA), then in the suspension of AuNPs for 20 min (sample name **S30/20**). The water-colloidal solution of AuNPs was prepared following the Turkevich protocol [21]. In the solution, all AuNPs were stabilized with trisodium citrate (further on abbreviated as citrate). The citrate molecules prevent AuNPs from self-agglomeration in water. The UV–vis spectra of the AuNP solution showed only one absorption band with *λ*_max_ = 520 nm, which corresponded to the LSPR band of single colloidal AuNPs of 17 nm diameter (see Figure S1 in the Supplementary Materials). No sign of agglomeration in the solution was detected.

We performed scanning electron microscopy (SEM) of the Si wafers with assembled AuNPs using a Gemini SEM (Zeiss, Germany). The wafers were attached to the sample holder using conductive double-sided graphite tape. The acceleration voltage was 3 kV. The AFM characterization of the wafers covered with AuNPs was done using a Bruker Multimode apparatus operating in the PeakForce^®^ mode in ambient conditions. The radius of the silicon cantilever (RTESPAW-300, Bruker, Germany) was 12 nm.

The XRD and the XRR studies were carried out using a PANalytical X′Pert Pro MRD XL (X`Pert, PANalytical B.V., Almelo, The Netherlands) equipped with a CuKα source of radiation (*λ* = 0.15406 nm). For the determination of the structural properties and phase composition of the films (Figure S2 in the Supplementary Materials) a W/Si parabolic mirror was used to create a high-intensity parallel beam. The diffracted beam was collimated by a parallel plate collimator with an acceptance angle 0.27°, used in combination with a 0.04 rad Soller slit. A four-bounce Ge(220) monochromator and 0.1 × 10 mm incident beam collimator were used for the XRR measurements. The diffracted beam was collimated by a parallel plate collimator with an acceptance angle 0.27°, used in combination with a 0.1 mm receiving slit (collimator slit). Such a combined X-ray investigation provided information about the composition, thickness, density, and roughness of the layer.

FTIR spectra of samples were recorded by a Perkin Elmer Spectrum One spectrometer in the frequency range 400–4000 cm^−1^. Reflection measurements were carried out using a single beam spectrometer UV–vis SpectraScan (ScanSci, Vila Nova de Gaia, Portugal). Specular reflectance spectra were recorded at an incidence angle of 45° for unpolarized light. An aluminum mirror was used as the calibration standard (Figure S3 in the Supplementary Materials). Modulation polarimetry was measured of the Q- and V-component of a Stokes vector in geometry external reflection at a 45° incident angle within the wavelength range *λ* = 400–1000 nm. The parameter ρ is a *Q*-component [42] that characterizes amplitude anisotropy of the interaction with light. The registered signal is the polarization difference *ρ*(*λ*) = *R*_s_−*R*_p_ [42,43,44]. *R*_s_ and *R*_p_ are the spectra of reflectance for s-polarized and p-polarized light, respectively.

## 3. Results and Discussions

### 3.1. Morphology Characterization

#### 3.1.1. Atomic Force Microscope (AFM)

**Figure 1:**
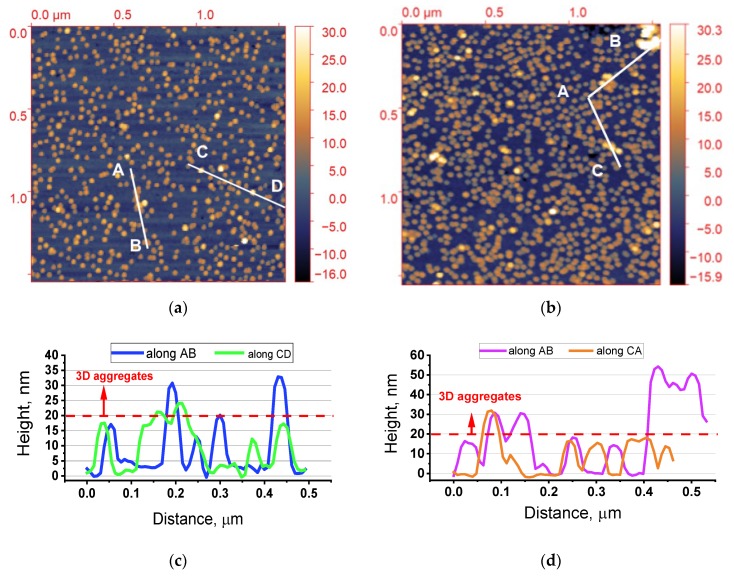
AFM images (1.54 × 1.54 μm^2^) of the Si wafer surface of (**a**) **S30** and (**b**) **S30/20** with corresponding profiles of cross-sections along A–B, CA shown in (**c**) and along A–B–C shown in (**d**). Each single round spot with a similar color profile corresponds to a single AuNP, while not-uniform objects or the ones with brighter contrast correspond to AuNP aggregates.

AFM revealed that the surface of **S30** and **S30/20** samples was covered mainly with single AuNPs homogeneously dispersed on the substrates (Figure 1). The average height (diameter) of AuNPs amounted to about 17 nm (Figure 1c). In addition to the single AuNPs, both samples contained aggregates with different sizes, shapes, and surface densities. Their density for **S30** was much lower than for **S30/20**. The average AuNP heights for **S30** did not exceed 30 nm, while for **S30/20** some were higher than 50 nm, as apparent from the cross-section along A–B (Figure 1c,d). Taking into account the average diameter of the AuNPs, we concluded that aggregates for **S30** are formed by two layers of AuNPs while on **S30/20** they may be formed by even three layers (Figure 1c). Distinguishing the internal structure of dimers and aggregates was not possible by AFM. Therefore, we performed in addition SEM studies.

#### 3.1.2. Scanning Electron Microscopy

**Figure 2:**
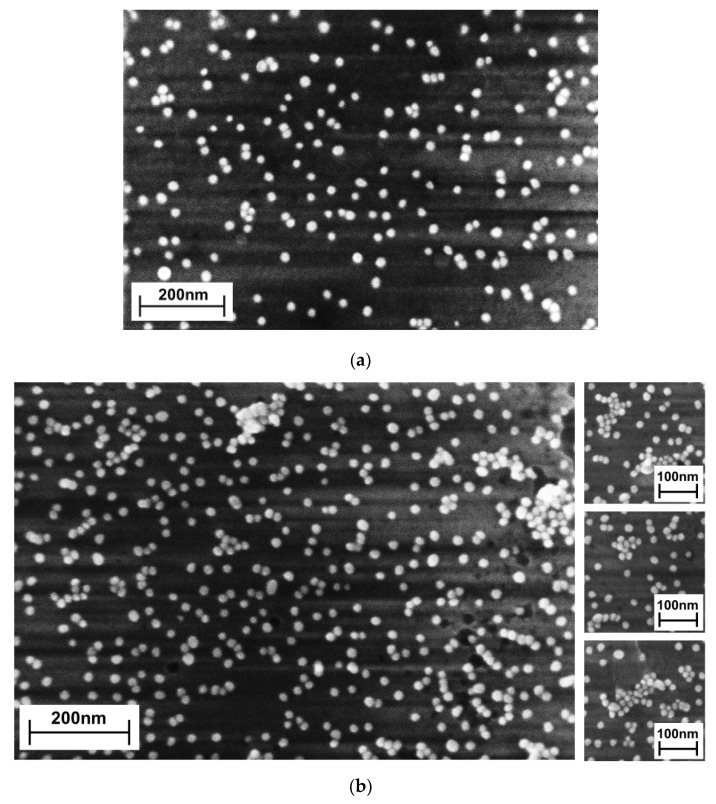
SEM images of (**a**) sample **S30** and (**b**) **S30/20**. The small panels on the right of (**b**) show further areas of sample **S30/20** with large aggregates.

The morphology of the assembly after the first processing step (**S30**) was characterized by arbitrarily located AuNPs on the Si wafer, as evidenced by SEM (Figure 2a). The majority of the nanoparticles were separated from each other to the distance of at least one nanoparticle diameter, which is explained by the presence of charged shells consisting of citrate molecules during the AuNP synthesis.

In addition to single AuNPs, some of them were self-organized in aggregates. The smallest were dimers (Figure 2a and Table 1). Larger aggregates contained from three to five nanoparticles self-assembled in lines or in nonlinear groups. The nature of the formation of aggregates on the APTES-coated surface can be explained in the following manner: The aggregation process occurs when AuNPs attach to the surface, since before that, all AuNPs exist as monomers in the colloidal solution, as proven by the absorption spectrum revealing just the single-particle plasmon peak at 520 nm. In the case of a glass surface, the aggregation effect is enhanced with the intrinsic roughness of the surface, which affects the quality of the APTES coating [20]. Therefore, when AuNPs attach to the regions with low quality of APTES coatings, a partial replacement of their citrate shells leads to the appearance of electrostatic (dipole–dipole) attraction between them. Our explanation correlates with the earlier observation that a partial replacement of citrate shells by thiols led to the formation of AuNP chains in solution [13]. Fifteen hours immersion of the APTES coated silicon surface in AuNP colloidal solution did neither lead to enlargement of the aggregates nor to a strong rise of their number (Supplementary Materials Figure S4a).

After the treatment of **S30** with HDT and immersion in the AuNP colloidal solution to give **S30/20**, the surface composition changed markedly. The number of single AuNPs increased by 69%, from 172 (**S30**) to 291 (**S30/20**) AuNP/μm^2^ (Table 1). The number of dimers almost doubled while the number of aggregates increased by a factor of five. In addition to the small aggregates made of three to five units in **S30**, much larger aggregates appeared in **S30/20** (Figure 2b). The SEM study of the **S30/20** surface (2 × 2μm^2^ in size) revealed that these large aggregates are composed of 10 to 40 AuNPs.

The comparison of the surface morphology of **S30** and **S30/20** (Table 1) suggests that one out of five large aggregates of **S30/20** may have originated from the initially formed aggregates of **S30**, so-called seeds. The other (second) type of seeds, which can also lead to AuNP aggregation, might have appeared during the moment of HDT contact with sample **S30**. We rationalize this as follows: During the first moment of HDT attachment, HDT molecules attack the surface of AuNPs, which is covered with negatively charged citrate molecules. Partial functionalization of AuNPs leads to a redistribution of AuNP surface charges and the appearance of uncompensated dipole moments. Therefore, if the AuNPs are close enough to each other before contact with HDT, they move towards each other, thereby forming aggregates by dipolar forces. This mechanism is responsible for AuNP chain formation in a colloidal solution [13]. This hypothesis is supported by the presence of elongated aggregates made up of four to seven AuNPs (Figure 2b) that were not detected before the addition of HDT (Figure 2a). Moreover, the majority of large extended aggregates (Figure 2b—right) had mainly a 2D structure with no or only very few AuNPs on top, as is apparent by AFM (Figure 1). This observation suggests that the process of assembly occurs mainly in the vicinity or particularly on the surface. Keeping in mind that AuNPs are anchored to the surface by static interactions of their negatively charged citrate shell and positively charged –NH_3_^+^ terminal groups of APTES, we do not exclude that they can “slide” over the surface in the presence of stronger dipole–dipole interactions. If this happens, one can expect that around large aggregates possessing strong, attractive forces, the surface density of single AuNPs should be diminished. This assumption is supported by the appearance of a large aggregates with the very low density of single AuNPs in their vicinity. This can even be observed by naked-eye (Figure S4b in the Supplementary Materials).

#### 3.1.3. X-Ray Reflectivity (XRR)

**Figure 3:**
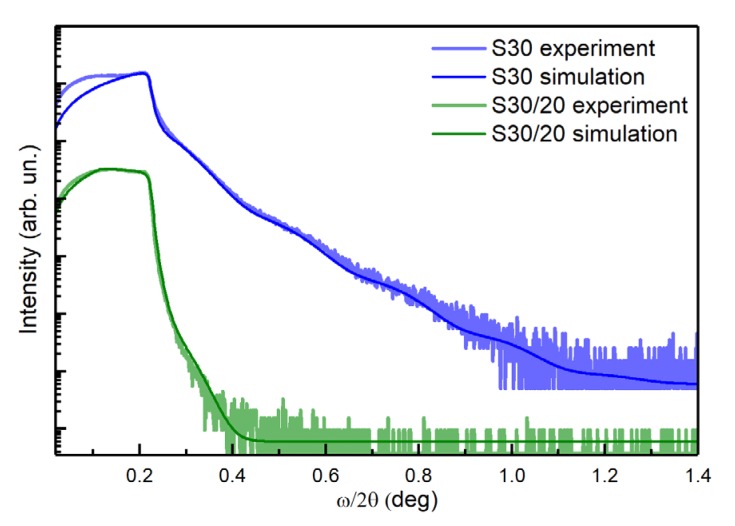
The XRR data obtained for sample **S30** with the inclusion of a small number of self-aggregated AuNPs and for sample **S30/20**. The experimental data were fitted with the X′Pert Reflectivity software package based on the Parratt formalism [45].

X-ray reflectivity analysis allowed the determination of the morphology, including thickness and roughness of the AuNP assembly on the entire Si wafer surface (Figure 3). The XRR of **S30** contained periodic intensity oscillations from which the thickness of an ensemble of nanoparticles could be determined. As can be seen from Figure 3, the attenuation rates of the curves for **S30** and **S30/20** were different, which carry information about the roughness. Thus, a rapid damping of oscillations (or their absence) and a substantial decrease in intensity indicated a significant roughness and, conversely, a weaker attenuation of the intensity indicated a small roughness.

Both spectra were fitted with the X′Pert Reflectivity software package based on the Parratt formalism [45]. The main fitting parameters were the concentration of gold in the composite layer (*x*), thickness, and roughness of the composite layer (*d* and *σ*). The concentration of gold directly correlates with the average density of the composite layer, which is determined by the critical angle.

$$\theta_{cr}=\sqrt{\frac{\lambda^{2}r_{el}}{\pi }\rho_{el}}$$

where *r_el_* is the classical radius of the electron, *ρ_el_* is the average electron density, and *λ* is the wavelength of the X-rays.

We used a model of a thick Si wafer and a thin composite layer (Au)*_x_*(HDT)_1−*x*_ to perform the spectra fitting. We used a single-layer model for **S30** and a two-layer model for the **S30/20** (for parameters see Table 2). The thickness of **S30** resulting from the fit agreed well with the average diameter of the AuNPs measured with SEM, AFM, and TEM (see Figure S1b in the Supplementary Materials). Therefore, we concluded that the chosen fitting approach using a two-layer model is suitable to study the morphology of **S30/20**.

Further fitting of the spectrum revealed (Figure 3) that the density of the upper layer of **S30/20** was significantly lower compared with the bottom layer. The size (~5 nm) of the layer roughness suggested that the number of 3D objects, which included aggregates and dimerized AuNPs with the orientation outward from the surface, was relatively low.

#### 3.1.4. Reciprocal Space Maps (RSM)

For comparing the morphology of **S30** and **S30/20** and for studying their influence on the diffuse X-ray scattering component, an intensity mapping using reciprocal space maps (RSMs) around the (000) reciprocal lattice point was measured.

The specular XRR profile (Figure 3) corresponded to the line on the RSM along the $q_{z}$ direction, for $q_{x}=0$. The diffuse scattering components ($q_{x}\neq 0$) were attributed to the roughness and to the lateral and vertical correlation lengths. A series of ω/2θ XRR scans at different incidence angles of X-rays was measured.

The reciprocal space map is the distribution of the intensity of the scattered X-rays as a function of coordinates in the reciprocal space, which are given by the following relations:

$$q_{x}=\left(\cos\left(\omega \right)-\cos\left(2\theta -\omega \right)\right)/\lambda \;q_{z}=\left(\sin\left(\omega \right)+\sin\left(2\theta -\omega \right)\right)/\lambda$$

where ω is the incidence angle, 2θ−ω is the scattering angle, λ is the wavelength of the X-rays, and *z* and *x* correspond to the axial directions along the normal and parallel to the surface, respectively.

**Figure 4:**
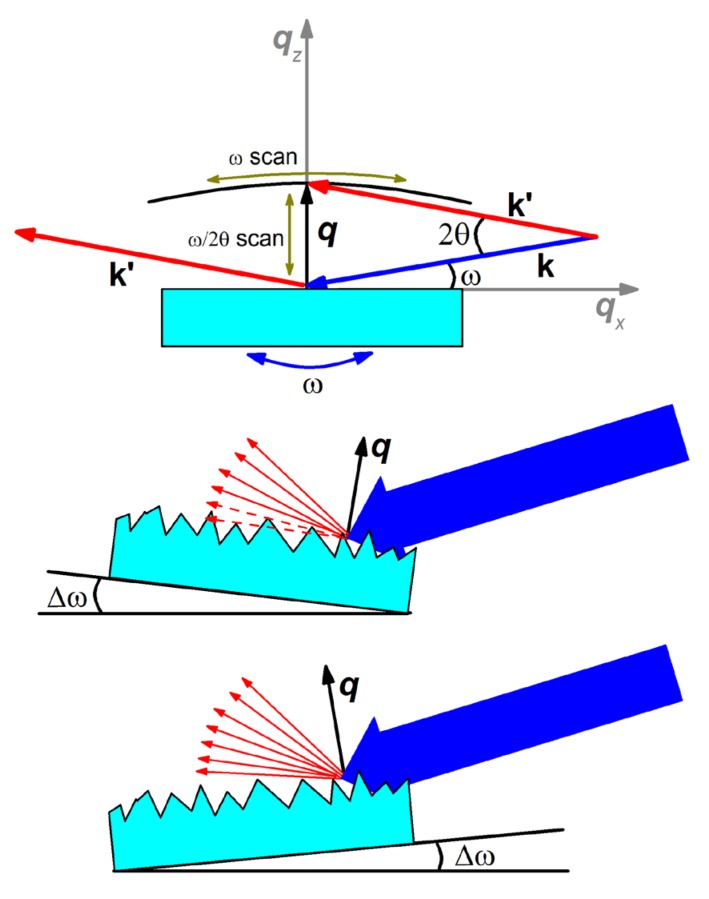
Schematic diagram of RSM measuring and formation scheme of diffuse scattering at different incidence angles of X-rays.

The diffuse scattering mechanism is sketched in Figure 4.

**Figure 5:**
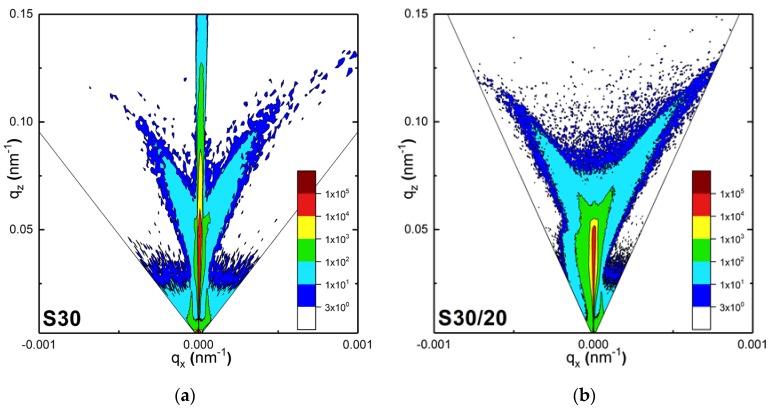
The experimental reciprocal space maps for (**a**) sample **S30** and (**b**) **S30/20**.

A significant contribution of the specular scattering component, manifested by a relatively high intensity along the $q_{z}$ direction at $q_{x}=0$, was observed for **S30**. This confirms our above observations about the low surface roughness of **S30**. For **S30/20,** practically all the incident intensities were scattered without a specular contribution to the RSM. Almost the entire intensity of the incident X-ray irradiation was scattered off surface inhomogeneities. This fact and the asymmetry of the diffuse scattering (Figure 5b) evidenced the presence of enhanced roughness in **S30/20**. Thus, the RSM indicated the absence of a vertical correlation of the roughness profiles [46], which suggested that all 3D objects in **S30/20**, including dimers and larger aggregates of AuNPs, were randomly distributed on the surface and did not have any particular orientation with respect to the surface.

### 3.2. Optical Characteristics of SAMs

#### 3.2.1. Fourier-Transform Infrared Spectroscopy (FTIR)

**Figure 6:**
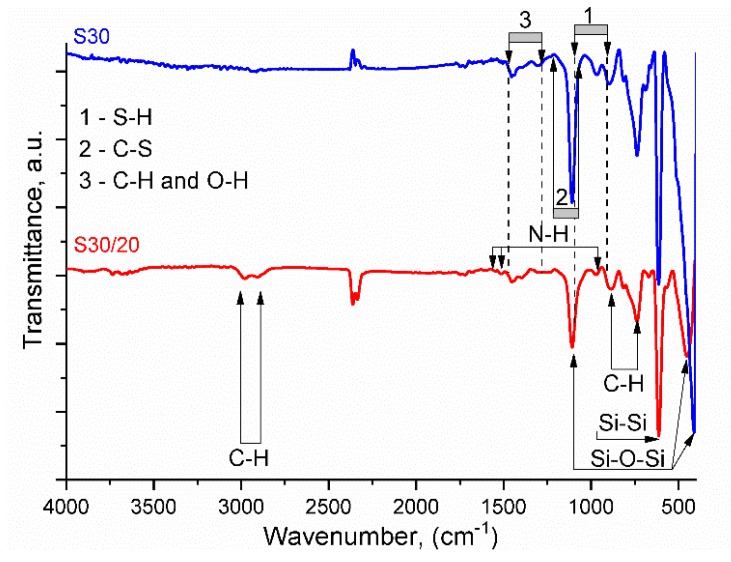
FTIR data for **S30** with a single AuNP layer with inclusion of minor number of self-aggregated nanoparticles in comparison **S30/20**).

The presence of organic layers on the Si wafer and the AuNPs was confirmed by FTIR spectroscopy measurements (Figure 6). For **S30** and **S30/20**, particularly intensive absorption at 620 cm^−1^ corresponded to vibrations of Si–Si bonds in the Si wafer. The presence of APTES in both samples stipulated identical bands in IR spectra at 410 and 460 cm^−1^, which are assigned to deformational vibrations of the Si–O–Si group [47]. Similarly, a sharp peak at 1115 cm^−1^ was attributed to asymmetric valence vibrations of the Si–O–Si group. Two pronounced bands at 744 and 890 cm^−1^ in both spectra were attributed to the rocking vibration of C–H bonds in the APTES layer. The amino groups caused a band at the intermediate wavenumber of 975 cm^−1^ corresponding to the rocking vibration of the N–H bond, and two weak bands at 1560 and 1515 cm^−1^ were attributed to the bending vibration of N–H. A series of weak vibrations in the range 1300–1450 cm^−1^ were attributed to deformation vibrations in C–H and O–H groups [48].

The overlapping of weak vibration of S–H bonds in the fingerprint range 900–1100 cm^−1^ as well as the overlap of vibrations of C–S bonds in the range 1200–1050 cm^−1^ were registered for **S30/20** with APTES and HDT organic components. However, two peaks at 2912 and 2994 cm^−1^ were typical of C–H stretching vibrations and indicate the HDT presence [48,49].

#### 3.2.2. Specular Reflectance of Non-Polarized Light

**Figure 7:**
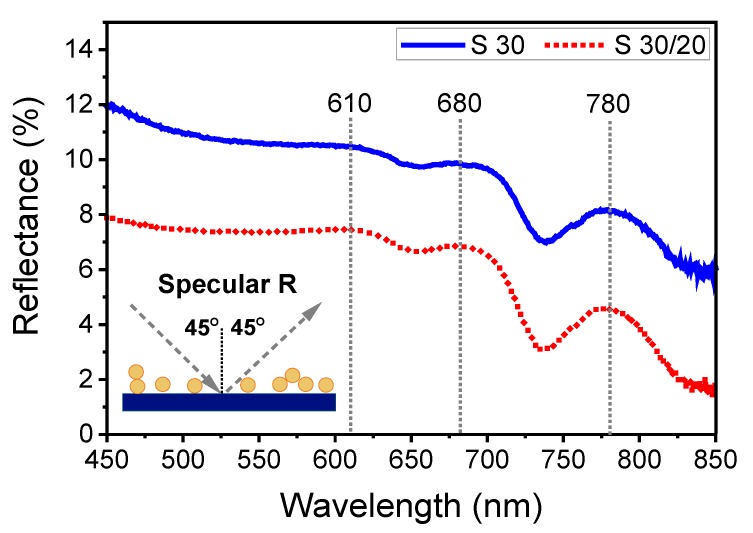
Optical reflectance spectra for sample **S30** (solid blue curve) and **S30/20** (red dotted curve). The incidence angle was 45°.

Figure 7 shows the specular reflectance spectra for samples **S30** and **S30/20**. The main difference between the spectra was their amplitude, which amounted to about 12% for sample **S30** and 8% for sample **S30/20**. Single, dimer, and more complex forms of AuNP aggregates were detected on Si wafers for **S30** and **S30/20** using AFM and SEM (Figure 1 and Figure 2). Therefore, it might have been expected that the light reflection spectra of non-polarized light would reveal unique distinguishable spectral characteristics for each type of nanoobjects on studied Si wafers. However, we observed a relatively low level of reflectance for both samples with a distinguishable difference of the amplitude for **S30** and **S30/20** (Figure 7). Such low reflectance could be associated with the resonant interaction of light with an array of NP, which was additionally reinforced by the light scattering effect (antireflective effects) [50]. Therefore, the amplitude of reflectance for **S30/20** was about 4% lower compared to **S30**, since light absorption and scattering increased for **S30/20** because of the higher surface density of AuNP compared to **S30** [51,52].

Furthermore, the spectra revealed several shallow and broad maxima and minima in the spectral range between 600 and 850 nm at very similar wavelength for both samples. To interpret these observations, we had to consider that several mechanisms, including absorption and scattering, determine the spectral dependence of the reflectance spectra of nanosystems on opaque substrates. [50,53,54]. Depending on the relative contribution of scattering an absorption, maxima as well as minima may occur at the wavelength corresponding to plasmonic excitations. Τhe contribution of scattering effects increases with increasing size and configuration of aggregates consisting of different numbers of single nanoparticles [53]. As a consequence, in the present measurement, the maxima in the reflectance spectra were associated with back-scattered light and LSPR dipolar mode excitation of NP arrays [50]. The minima in the spectra could be associated with the excitation of higher multipole modes (quadrupolar) and forward-scattered light [50].

The profiles of the reflectance spectra of both samples were rather similarly corresponding to the similar composition of **S30** and **S30/20**, i.e., both samples contained monomeric AuNPs, dimers, and aggregates with multiple forms (Figure 1 and Figure 2). It might have been expected that 3D aggregates of **S30/20** would give more impact on the resulting spectrum. However, as it was shown for multilayered AuNP assemblies, some of the plasmon modes can vanish [55].

The LSPR for single AuNPs was expected near 520 nm [56], since the absorption spectra of our colloidal AuNPs exhibited a peak at 520 nm in solution (Figure S1 in the Supplementary Materials). The position of the LSPR may have been slightly shifted due to the difference in the dielectric constant of water (*ε* = 1.77) and air (*ε* = 1), when single AuNPs were deposited on glass and measured in transmission in air [20,56]. Therefore, on the Si wafer in the reflection geometry, the position of the LSPR also may slightly vary. However, there was no indication of a peak around 520nm on any of the samples, despite their presence on the surface, as revealed by SEM (Figure 2). The absence of such characteristic peak may have several reasons. At first, for aggregates consisting of individual spheres and arrays, simultaneously the dipolar and quadrupolar modes may be excited and interfere in the scattered light [57]. As a result, the spectral contours of these modes may overlap and eventually cancel. Additionally, according to [58,59], interband transitions of AuNPs affect the shape of the LSPR spectrum. All features of specular reflectance spectra for **S30** and **S30/20** are related to simultaneous resonant interaction between the single NPs and various forms of aggregates with electromagnetic radiation. Mainly, such an interaction takes place between single AuNPs interconnected in dimers with larger aggregates and different interparticle distances, as were detected by morphological investigations. The electromagnetic fields related to the LSPR mode of one NP may then influence the response of neighboring nanoparticles. This electromagnetic coupling can take several forms: via near-fields and via far-fields [60]. Particles can interact via near-field coupling when they are relatively densely packed, leading to significant spectral shifts of the plasmon resonances and modification and splitting of their line-shapes due to the hybridization of the plasmon modes.

Variations of the reflectance become more apparent with increasing the size and configuration of nanoobjects, since the level of hybridization of plasmon modes of interconnected AuNP increases [61,62]. We attributed the broad band around 610 nm to the reflectance of dimers and had two main origins. It may have appeared as the result of the coupled dipole oscillation of the longitudinal optical excitation mode along the dimer axis between the two nanoparticles [31]. At the same time, the dipole–dipole interactions within AuNP agglomerates may have given an additional impact on the formation of the maximum [61]. Both of these types of coupling create new collective oscillation modes. Besides, **S30/20** may have contained two types of dimers. The first one appeared due to self-aggregation during the first immersion, while the second one was interconnected via HDT. Consequently, instead of a single narrow reflection mode [31], we observed a broad hill at around 610 nm. Similarly, the maxima around 680 and 780 nm could be attributed to hybridization modes of trimers and more complex objects, e.g., chains [62,63] based on the same mechanisms as the dipole mode.

As mentioned above, also the minima may reflect plasmon excitations, in particular if the absorption dominates over the scattering contribution. We therefore tentatively attribute the minima around 660 nm and 730 nm as quadrupular modes of dimers and trimers, respectively.

#### 3.2.3. Modulation Polarimetry

**Figure 8:**
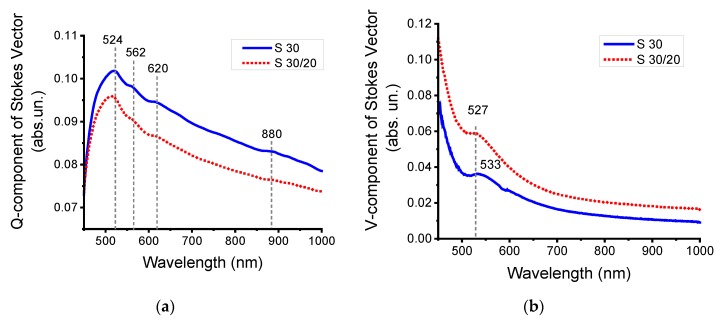
The modulation polarimetry spectra for samples **S30** and **S30/20**: (**a**) amplitude and (**b**) phase anisotropy.

The spectral characteristics of *Q*- and *V*-components of the Stokes vector were measured to reveal polarization-dependent amplitude and phase anisotropy effects in the external reflection for plasmonic NP arrays (Figure 8). We used a method based on recording the amplitude difference in the reflectance intensity between *s*- and *p*-polarized beams, which suppressed the common-mode noise. The registration signal of the *Q* component of the Stokes vector is larger and more informative than that of the method of interaction with only p-polarized light [20].

The *Q*-component takes into account the simultaneous interaction with *s*- and *p*-polarized electromagnetic radiation. The *Q*-component is determined by the linear (amplitude) anisotropy since it is equal to the difference between the reflectance of *R*_s_ and *R*_p_. The decrease of the spectral amplitude of *Q*(*λ*) for **S30/20** was related to an increase of nanoparticle concentration and a resonance interaction with electromagnetic radiation [42,64] (Figure 8a).

The observed spectral characteristics for plasmonic nanoparticle arrays originate from the differential excitation of the two orthogonal dipolar plasmon resonances (transverse and longitudinal) by orthogonal linear polarization [65]. For oblique incidence of light of both s- and p-polarizations on nanoparticle arrays, the in-plane and out-of-plane resonances that characterize excitations of plasmon oscillations in the plane of the array and perpendicular to the array plane are excited [66]. In this case, the polarization-dependent light interaction with the NP array leads to hybridization between parallel and orthogonal surface lattice resonances [67].

Additionally, for disordered arrays, the broken symmetry could lead to the in-plane excitation of a quadrupolar mode and a change of extinction or reflectance [68]. The parameter *V*(*λ*) is determined by the circular (phase) anisotropy, since it contains the phase difference between orthogonal polarization states, *s*- and *p*-, correspondently [69]. The spectral characteristics of the *V*-component of the Stokes vector, on the other hand, characterize the resulting phase between the competing dipolar or hybridized plasmon modes [65].

The features of *V*(*λ*) for **S30** and **S30/20** in the 500–620 nm spectral range are related to the LSPR and has a maximum at 527/533 nm since the rotation of the polarization state in circular anisotropy is always sensitive to the localization of light and LSPR [70].

An increase in the number of particles of various shapes on the surface of silicon wafers leads to a decrease in the amplitude of the components of the Stokes vector (*Q* and *V*) of reflected light due to the excitation of localized plasmon resonance. Monomers, dimers, trimers, and aggregates contribute to this integral change in the amplitude of the components of the Stokes vector.

## 4. Conclusions

The combination of Χ-ray techniques, modulation polarimetry, FTIR, AFM, and SEM methods allowed an in-depth analysis of the morphology of sophisticated organometallic coatings made of AuNPs cross-linked with 1,6-hexanedithiol molecules directly on a silicon wafer. We found that the AuNP dimers were formed not only through the dithiol molecules, as expected, but could appear as a result of self-aggregation directly on the APTES-coated wafer surface. For distinguishing the difference in optical signals from self-aggregated dimers and the ones formed through dithiols, further studies are necessary.

In addition to the self-dimerized AuNPs, aggregates with non-uniform shapes were detected. The small lateral size of these aggregates, after the first incubation of the APTES-coated wafer in the colloidal solution, suggests that they can appear due to local defects in the APTES coating. The second incubation led to their significant enlarging. Meantime, the number of dimers remained rather low. These observations suggest that aggregates on the surface attract NPs from the solution more efficiently than the single NPs. The nature of the force that drives this aggregation could not be revealed evidently yet. One possibility could be a net dipole moment of the aggregates, which may attract citrate-coated AuNP from the solution more efficiently than single AuNPs.

Despite the presence of aggregates during both stages of precipitation, the polarized light spectroscopy revealed a strong signal from AuNP dimers. The feasibility of modulation–polarization spectroscopy for the detection of plasmonic modes for the single, dimer, and larger aggregates of NPs was illustrated for the simple plasmonic system of an AuNp assembly.

## Figures and Tables

**Table 1:** Statistical data obtained from the SEM images Figure 2a,b.

Types of Nanoobjects	Sample S30, per 1 μm^2^	Sample S30/20, per 1 μm^2^	Increase Rate, %
Monomers	172	291	69
Dimers	16	45	180
Agglomerates	8	49	512

**Table 2:** Parameters of the samples measured with XRR.

Sample	*ρ*, g/cm^3^	Au, % vol.	Thickness d, nm	Roughness *σ*, nm
**S30**	1 layer: 2.08	6 ± 1	18 ± 2	1.3 ± 0.1
**S30/20**	1 layer: 1.17 2 layer: 2.19	1 ± 1 6.6 ± 1	- -	5 ± 1

## Supplementary Materials

The following are available online at https://www.mdpi.com/2079-4991/10/3/512/s1, Figure S1 UV-vis spectrum and analysis of AuNPs, Figure S2: XRD patterns of the samples **S30** and **S30/20**, Figure S3: Reflectance spectrum of Al mirror, Figure S4: SEM images of control samples.
