# Supplementary material for: Gold Nanoparticle Self-Aggregation on Surface with 1,6-Hexanedithiol Functionalization
Source: Nanomaterials (Basel). 2020 Mar 11;10(3):512. doi: 10.3390/nano10030512 (PMC7153467; doi:10.3390/nano10030512)
Supplement: Supplementary file 1 [file nanomaterials-10-00512-s001.pdf]

nanomaterials

SUPPLEMENTARY INFORMATION

Gold Nanoparticle Self-Aggregation on Surface with 1,6-Hexanedithiol Functionalization

Maksym Stetsenko[1,2], Tetiana Margitych[3], Serhii Kryvyi[2,4], Lidia Maksimenko[2], Ali Hassan[1], Svitlana Filonenko[5], Baikui Li[1,*], Junle Qu[1,*], Elke Scheer[6], Sergii Snegir[6*]

[1] Key Laboratory of Optoelectronic Devices and Systems of Ministry of Education and Guangdong Province, College of Physics and Optoelectronic Engineering, Shenzhen University, 518060, Shenzhen, PR China; stetsenkomax@gmail.com (M.S), 15alirao@gmail.com (A.H.)
[2] V. Lashkaryov Institute of Semiconductor Physics, National Academy of Sciences of Ukraine, 03680, Kyiv, Ukraine; maximenko_lida@ukr.net (L.M.)
[3] Kyiv Institute for Nuclear Research, National Academy of Sciences of Ukraine, 03680, Kyiv, Ukraine; margtanya@gmail.com (T.M.)
[4] Institute of Physics, Polish Academy of Sciences, Warsaw, Poland; serkriviy@gmail.com (S.K.)
[5] Pisarzhevski Institute of Physical Chemistry, National Academy of Sciences of Ukraine, 31 Prospect Nauky, 03028 Kyiv, Ukraine; svitmail@ukr.net
[6] University of Konstanz, Department of Physics, Universitätsstraße 10, 78464 Konstanz, Germany; Elke.Scheer@uni-konstanz.de (E.S.)
[*] Correspondence: sergii.snegir@uni-konstanz.de (S. Snegir), libk@szu.edu.cn (B. Li), jlqu@szu.edu.cn (J. Qu)

- UV-vis light absorption colloidal solution
- X-ray diffraction
- Plasmon hybridization model

UV-vis light absorption colloidal solution

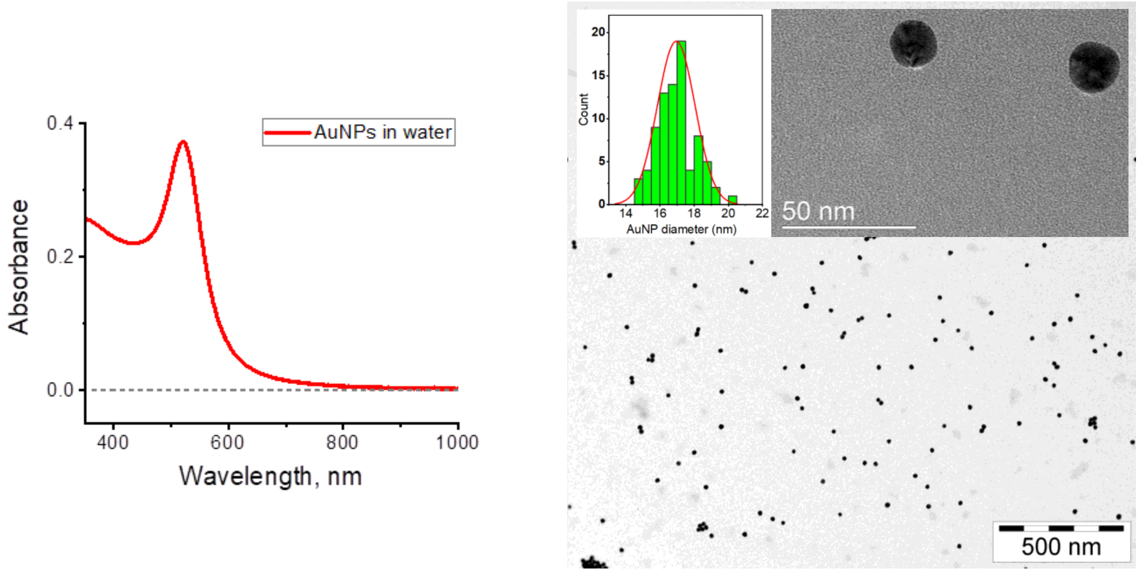

(a) (b)

Figure S1. (a) UV-vis light absorption spectra of AuNPs synthesized according to Turkevich protocol. The adsorption band (520nm) corresponds to the LSPR of single AuNPs in solution. (b) TEM image of AuNPs with corresponding histogram of their size distribution and inserted TEM image of two separate AuNPs with large magnification, representing spherical-like shape of AuNPs.

X-ray diffraction

The XRD of AuNP assemblies was in good agreement with that of bulk gold and showed no evidence for contamination or the formation of any secondary gold phase [1]. The spectra display a pronounced peak at 38.3°, which we attributed to Au(111) planes parallel to the surface of the silicon substrate. The second peak at 44.4° with the weaker relative intensity we attributed to Au(200).

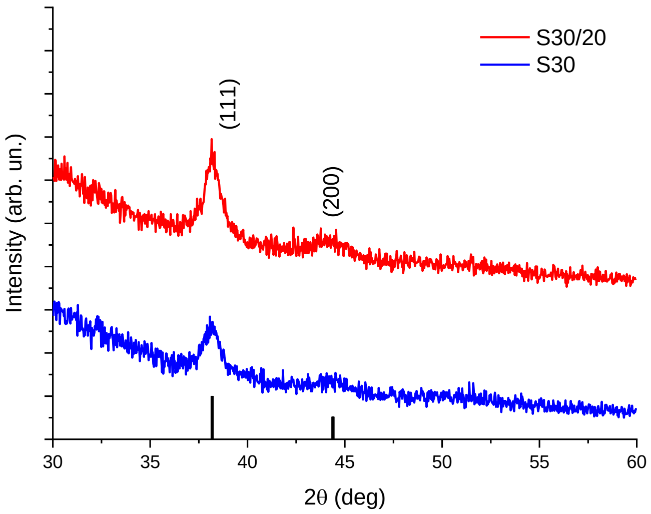

Figure 2S. XRD patterns of the samples **S30** and **S30/20**

The peaks (111) were fitted using the Pseudo-Voigt function and positions of the peak centers were obtained. Then, using the Bragg equation, lattice parameters for gold nanoparticles are determined. The XRD data of these samples are summarized in Table S1.

Table S1. X-ray diffraction data

Sample	a, Angstrom	delta a, Angstrom	FWHM 2Teta, deg	Scherrer size, Angstrom
S30	4.0879	0.008	1.03	81.59507499
S30/20	4.0815	0.011	1.19	70.63733596

The size obtained from Scherrer's formula [2] is the average or apparent crystallite size and is not necessarily the same as the particle size because the effects of aggregation are not taken into account in this formula[3]. The shift of the peaks (111) on the diffraction patterns (Fig. 2) indicates a change in the lattice parameter of the material of the studied films. The effect of reducing the lattice parameter may be explained by the presence of surface tension forces in the NP [4]

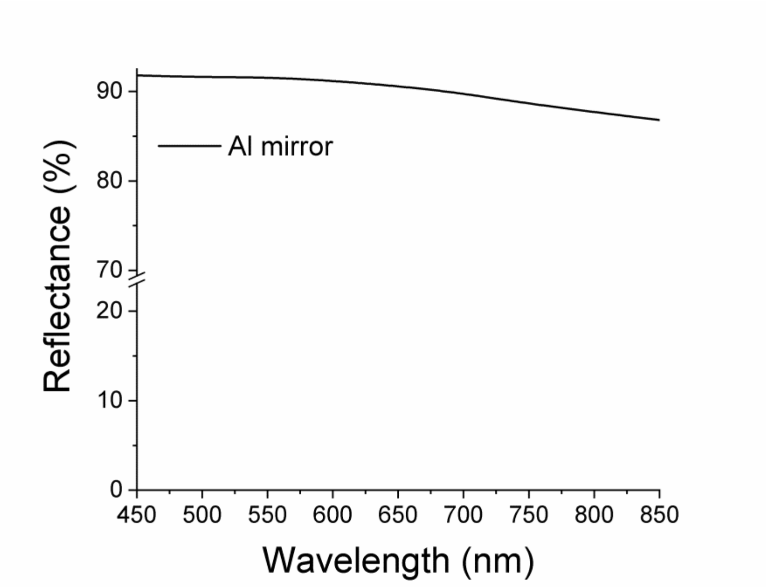

Figure S3. Reflactance spectra of Al mirror (reflactance standart).

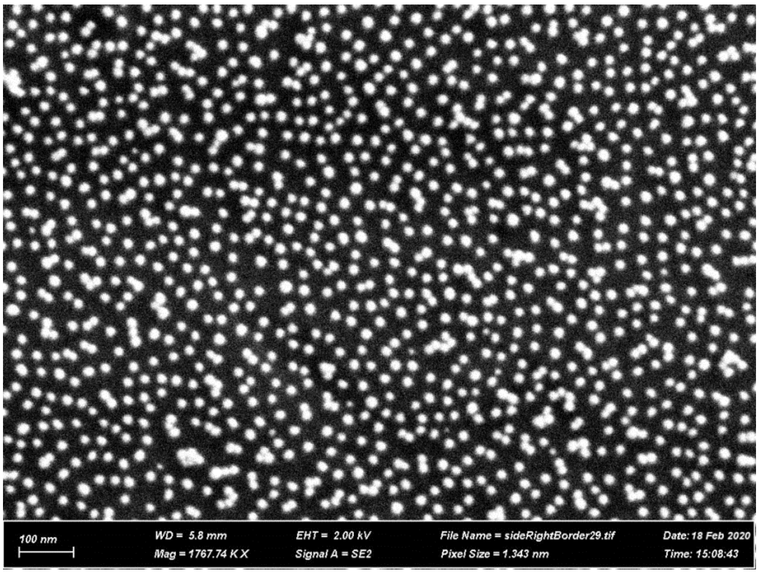

(a)

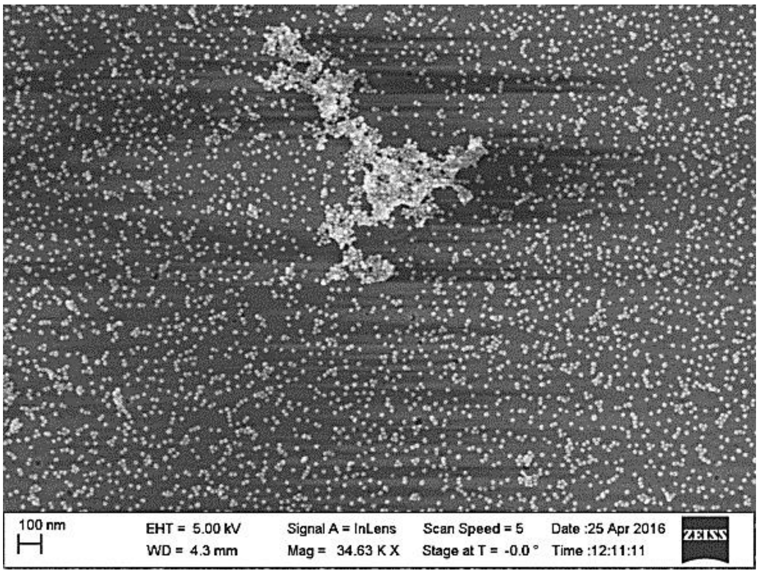

(b)

.

Figure S4. (a) - SEM image of the sample S15H, which was coated with APTES in a similar manner as S30 but immersed in AuNP water colloidal solution for 15 hours, **(b) -** SEM image of the sample S20/30. The surface contains mainly aggregates with the lateral site about 100nm. The size of the largest which was detected, was about 1µm. The density of single NP in the distance of about 300nm around the large aggregate is rather low, compared to the density far from the aggregate than 300nm.

Table S1. Statistical data obtained from the SEM images Fig. 2 a) and Fig. S2a)

Types of nanoobjects	Sample S30, per 1 μm^2	Sample 15H, per 1 μm^2
Monomers	172	625
Dimers	16	78
Agglomerates	8	48

Reference

1. Petkov, V.; Peng, Y.; Williams, G.; Huang, B.; Tomalia, D.; Ren, Y. Structure of gold nanoparticles suspended in water studied by X-ray diffraction and computer simulations. *Phys. Rev. B* **2005**, *72*, 195402, doi:10.1103/PhysRevB.72.195402.
2. Scherrer, P. *Bestimmung der Größe und der inneren Struktur von Kolloidteilchen mittels Röntgenstrahlen. Nachrichten von der Gesellschaft der Wissenschaften zu Göttingen, Heidelberg Mathematisch-Physikalische Klasse*; Springer: Berlin, Germany, 1918; pp. 387–409, doi:10.1007/978-3-662-33915-2_7.
3. Jensen, H.; Pedersen, J.H.; Jrgensen, J.E.; Pedersen, J.S.; Joensen, K.D.; Iversen, S.B.; Sgaard, E.G. Determination of size distributions in nanosized powders by TEM, XRD, and SAXS. *J. Exp. Nanosci.* **2006**, *1*, 355–373, doi:10.1080/17458080600752482.
4. Solliard, C.; Flueli, M. Surface stress and size effect on the lattice parameter in small particles of gold and platinum. *Surf. Sci.* **1985**, *156*, 487–494, doi:10.1016/0039-6028(85)90610-7.
